# The Critical Transition in Surgical Experience: Impact of the Early Years of Surgical Practice on Perioperative Outcomes and Team Collaboration

**DOI:** 10.1002/wjs.70030

**Published:** 2025-08-01

**Authors:** Wongsakorn Chaochankit, Seechad Noonpradej, Srila Samphao, Somrit Mahattanobon, Chutida Sungworawongpana

**Affiliations:** ^1^ Division of General Surgery Department of Surgery Faculty of Medicine Prince of Songkla University Songkhla Thailand; ^2^ Department of Anesthesiology Faculty of Medicine Prince of Songkla University Songkhla Thailand

**Keywords:** ASA classification, Clavien–Dindo classification, in‐hospital mortality, mentorship, surgical experience, surgical outcomes

## Abstract

**Background:**

Surgical experience and team structure play critical roles in determining perioperative outcomes. The early years of surgical practice represent a transition phase during which mentorship and case selection are essential to optimize patient safety while developing technical skills.

**Methods:**

A retrospective cohort study was conducted to analyze 1123 surgeries performed by a single surgeon alone (S), in collaboration with a senior co‐surgeon (S + S), or with a junior co‐surgeon (S + J) during the first five years of independent practice. Perioperative parameters and postoperative outcomes were compared across groups. Multivariate Cox regression was used to identify risk factors associated with high Clavien–Dindo complications (grade > 2), in‐hospital mortality, and overall mortality.

**Results:**

Surgeries in the S + S group involved the most complex cases, with significantly longer operative times, greater blood loss, and higher rates of complications and mortality. Multivariate analysis identified upper GI surgery, high ASA class, emergency surgery, and malignancy as independent predictors of adverse outcomes. In contrast, laparoscopic surgery and breast procedures were protective. Importantly, surgeons with < 3 years of experience had significantly higher overall mortality risk (HR 2.73, *p* < 0.001). The S + J group demonstrated intermediate outcomes, suggesting that junior involvement under appropriate supervision maintains patient safety while supporting skill development.

**Conclusion:**

The first 3 years of surgical practice represent a critical learning phase. Although complication rates decrease with experience, increased case complexity in later years requires ongoing support. Structured mentorship and progressive case exposure are key to ensuring safe and effective transitions from trainee to independent surgeon.

## Graphical abstract

Surgical experience and team structure play critical roles in determining perioperative outcomes. The early years of surgical practice represent a transition phase during which mentorship and case selection are essential to optimize patient safety while developing technical skills. A retrospective cohort study was conducted to analyze 1123 surgeries performed by a single surgeon alone (S), in collaboration with a senior co‐surgeon (S + S), or with a junior co‐surgeon (S + J) during the first five years of independent practice.

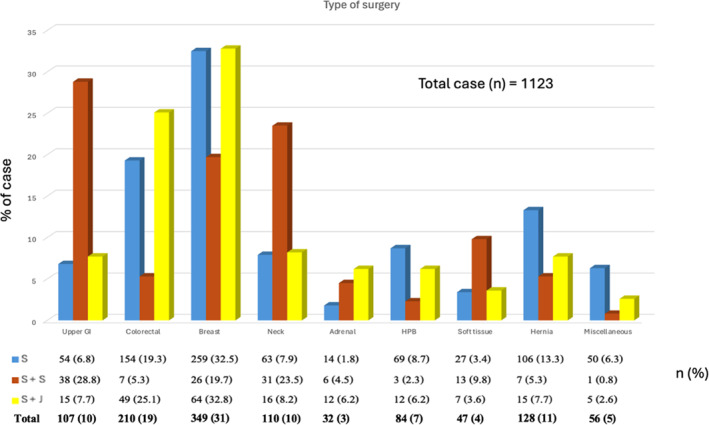

## 1 Background

Surgical outcomes are influenced by various factors, including patient characteristics, surgical techniques, and perioperative management [1]. Experience is a critical determinant of surgical success. Reportedly, improved outcomes, reduced complications, and lower mortality rates are guaranteed for patients when surgeons have more experience [2, 3]. However, the effect of collaboration between surgeons with different levels of experience has not been thoroughly investigated.

Surgical collaboration plays a crucial role in optimizing outcomes, especially for complex procedures [4]. Experienced surgeons have advanced technical skills, sound judgment, and the ability to manage intraoperative complications. Their familiarity with anatomy, instruments, and hemodynamics allows for timely, precise decision‐making [5]. Importantly, their mentorship helps junior surgeons develop both technical and cognitive competencies within a supportive learning environment [6]. This supervisory team structure contributes to surgical safety, continuity of best practices, and improved operating room performance [7].

The length of experience required for an individual to be considered a senior surgeon varies based on the specialty and healthcare system. In Thailand, this typically includes medical school, residency, and often fellowship, followed by several years of independent practice [8]. Although the benefits of surgical experience are well recognized, the specific impact of mentorship and senior–junior collaboration on outcomes remains underexplored [4, 7, 9, 10, 11]. Few investigations have assessed whether mentorship improves precision, reduces complications, or introduces communication barriers [2, 3]. Existing research often focuses on individual surgeon performance rather than team dynamics in complex procedures [12, 13]. Additionally, most studies were conducted in Western settings, limiting generalizability to other training systems [11]. The long‐term implications of transitioning from junior to senior roles, as well as the benefits and trade‐offs of collaborative versus solo practice, warrant further exploration [9, 10].

Therefore, this study specifically analyzes the operative experience of a single early‐career surgeon over the first 5 years of independent practice to minimize variability and better examine individual progression and mentorship impact.

## 2 Methods

This retrospective cohort study was designed to evaluate the impact of surgeon experience and collaboration on perioperative outcomes using the information system of the Songklanagarind Hospital. The data of patients who underwent surgery in the major operative theater at Songklanagarind Hospital between August 2019 and October 2024 (the first 5‐year work of a new surgeon) were retrospectively collected from an operative registry, in which data are prospectively collected. We included patients who underwent surgery conducted by a single primary surgeon and team collaboration in the first 5 years of their career experience. All included cases were performed by the same board‐certified general surgeon between August 2019 and October 2024. We excluded patients who underwent procedures in the minor operative theater under local anesthesia, as well as those without complete medical records in the hospital information system. The Ethics Committee of the Prince of Songkla University approved the study protocol. Data, including demographic details, clinical history, and perioperative parameters, were obtained from patient records. The dataset comprised variables, such as patient age, sex, history of cancer, surgical risk (low‐, intermediate‐, and high‐risk), type of surgery performed, nature of the operation (open vs. minimally invasive), operative time, estimated blood loss, type of anesthesia administered, American Society of Anesthesiologists classification, emergency case status, year of surgery, intensive care unit (ICU) admission, length of hospital stay, postoperative complications, Clavien–Dindo classification, and in‐hospital mortality.

### 2.1 Variables and Definitions

The following definitions were applied to standardize data interpretation across all cases:

– Mentorship was defined as preoperative planning and intraoperative decision‐making assistance provided by another surgeon without assuming the role of primary operator.
– Surgical complexity was inferred based on a combination of procedure type, ASA classification, operative time, and estimated blood loss (EBL) quartiles.
– Cancer history included patients with active malignancy, those undergoing oncologic surgery, or those treated for cancer within the past 5 years.

To examine the effect of surgeon collaboration on perioperative outcomes, patients were categorized into three distinct groups based on the composition of the surgical team.

1. Single Surgeon (S): Here, a single surgeon performed the entire procedure independently without being assisted by a co‐surgeon.
2. Single with Senior Surgeon (S + S): In this category, the single surgeon was assisted by a senior co‐surgeon who had > 10 years of surgical experience. Senior surgeons play an active role in the procedure, providing technical expertise and decision‐making skills.
3. Single with Junior Surgeon (S + J): Here, a junior surgeon assisted the single surgeon. Junior surgeons were classified as less experienced and were typically in the early stages of independent practice. This collaboration allows junior surgeons to gain hands‐on experience under the guidance of a more experienced lead surgeon.

Using this classification, we explored whether involving senior or junior cosurgeons influences key perioperative outcomes, including surgical efficiency, complication rates, and recovery. Data were analyzed to compare outcomes between groups and evaluate factors such as operative time, blood loss, hospital stay, and complications. Statistical tests determined the significance of variations, with adjustments for confounders such as comorbidities and procedure complexity. Surgeon team composition was determined during preoperative scheduling based on anticipated complexity, urgency, and availability. Cases in which a senior or junior surgeon joined intraoperatively in an unplanned manner were excluded from the subgroup analysis.

### 2.2 Statistical Considerations

Baseline characteristics were summarized using medians and interquartile ranges for continuous variables, and frequencies with percentages for categorical variables. Group comparisons used chi‐square or Fisher's exact tests for categorical data, and Student's t‐test or Kruskal–Wallis test for continuous data, depending on distribution. To evaluate postoperative outcomes—including high‐grade Clavien–Dindo complications (grade > 2), in‐hospital mortality, and overall mortality—we performed separate multivariate Cox proportional hazards models for each outcome to avoid conflating distinct clinical endpoints. Each model was adjusted for key indicators of patient and surgical complexity, including procedure type, ASA classification, emergency status, operative time, and estimated blood loss (EBL). Hazard ratios (HRs) with corresponding 95% confidence intervals (CIs) and *p*‐values were reported to identify independent associations. No composite outcome was used in this analysis.

All analyses were performed using R version 3.6.3 (R Foundation for Statistical Computing, Vienna, Austria), with a two‐sided *p*‐value < 0.05 considered statistically significant. This statistical approach ensured a detailed and reliable assessment of how surgeon experience, collaboration models, and perioperative risk factors influence surgical outcomes.

## 3 Results

**FIGURE 1:**
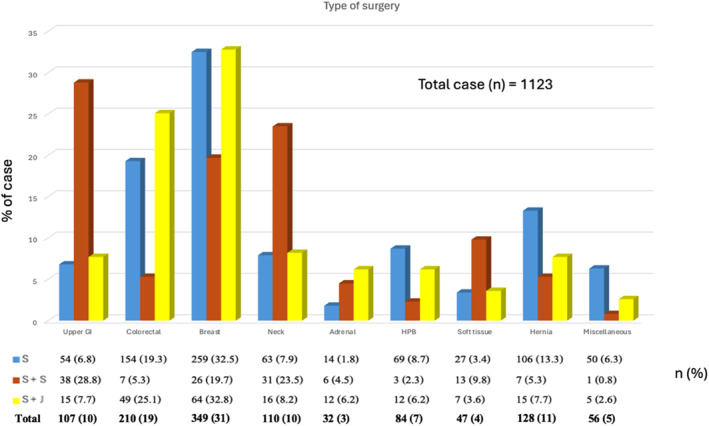
Relationship among case percentage, type of surgery, and surgeon collaboration.

The study analyzed 1123 cases, divided into single surgeon (S, 70.9%), senior‐supervised (S + S, 11.7%), and single with junior (S + J, 17.4%) teams. Notable differences in case complexity and perioperative outcomes were found. The S + S group had the highest cancer prevalence (53.8%), most open surgeries (87.1%), longest operative time (median 182.5 min), and greatest blood loss (median 50 mL). In contrast, the S and S + J groups handled fewer complex cases, with shorter operative times and lower blood loss. Regarding postoperative outcomes, the length of hospital stay (LOS) was the longest in the S + S group (median: 5 days), followed by the S + J (4 days) and S (3 days) groups. Additionally, mortality was significantly higher in the S + S group (4.5%) than that in the S (0.9%) and S + J (1.0%) groups, emphasizing that senior surgeons are often assigned the most critical and high‐stakes cases (Table 1). Complex procedures, particularly open and oncologic surgeries, were predominantly performed in the S + S group, reflecting the tendency for senior surgeons to manage higher‐risk cases. In contrast, the S + J group was more frequently assigned to cases of lower surgical complexity. These findings highlight the importance of a structured mentorship framework that allows junior surgeons to progressively engage with more challenging procedures under the supervision of experienced colleagues (Figure 1). Analysis of surgical collaboration over 5 years showed evolving roles with growing experience. By the third year, a shift toward S and S + J cases emerged, indicating progression in training. Only data from the first 5 years were analyzed due to missing data (see Table S1). Postoperative complications varied by collaboration model. S + S cases had the highest rates, likely due to case complexity. S + J cases showed intermediate rates, suggesting effective supervision. Solo surgeries had the lowest rates, likely because experienced surgeons handled lower‐risk cases (see Table S1). Among 79 patients with Clavien–Dindo > 2 complications, rates increased with surgical risk: 1.6% (low‐risk), 11.5% (intermediate), and 34.7% (high‐risk). Complication rates by surgeon experience fluctuated—highest in year 1 (12.1%), dropping in year 2 (7%), rising in year 3 (11.3%), reaching a low in year 4 (2%), and increasing again in years 5–6 (8% and 6%), likely reflecting growing independence and case complexity (see Table S1).

**TABLE 1:** Baseline characteristics of patients (*n* = 1123).

Variable	Total 1123	S 796 (70.9%)	S + S 132 (11.7%)	S + J 195 (17.4%)	*p* value
Age (median, IQR)	57	57 (46, 66)	56.5 (44.8, 66.2)	59 (49, 68.5)	0.19^a^
Male (*n*, %)	447 (40)	320 (40.2)	54 (40.9)	73 (37.4)	0.75^b^
Cancer (*n*, %)	378 (33.7)	224 (28.1)	71 (53.8)	83 (42.6)	< 0.001^b^
Risk of surgery (*n*, %) Low Intermediate High	676 (60.2) 375 (33.4) 72 (6.4)	501 (62.9) 265 (33.3) 30 (3.8)	65 (49.2) 43 (32.6) 24 (18.2)	110 (56.4) 67 (34.4) 18 (9.2)	< 0.001^b^
Open surgery (*n*, %)	798 (71.1)	563 (70.7)	115 (87.1)	120 (61.5)	< 0.001^b^
Operative time (median, IQR)	125	120 (80, 170)	182.5 (120, 291.2)	140 (95, 230)	< 0.001^a^
EBL (median, IQR)	20	10 (5.50)	50 (13.8, 200)	20 (5100)	< 0.001^a^
General anesthesia (*n*, %)	998 (88.9)	691 (86.8)	126 (95.5)	181 (92.8)	0.006^b^
ASA classification (*n*, %) I II III IV V	53 (4.7) 772 (68.7) 283 (25.2) 13 (1.2) 2 (0.2)	34 (4.3) 557 (70) 193 (24.2) 11 (1.4) 1 (0.1)	10 (7.6) 77 (58.3) 42 (31.8) 2 (1.5) 1 (0.8)	9 (4.6) 138 (70.8) 48 (24.6) 0 (0) 0 (0)	0.101^b^
Emergency (*n*, %)	165 (14.7)	137 (17.2)	12 (9.1)	16 (8.2)	< 0.001^a^
ICU (median, IQR)	4.5	4 (3, 9.5)	4.5 (3, 5.8)	5.5 (3.5, 7.5)	0.697^b^
LOS (median, IQR)	3	3 (3, 5)	5 (3, 10)	4 (3, 6)	< 0.001^a^
In hospital death (*n*, %)	15 (1.3)	7 (0.9)	6 (4.5)	2 (1)	0.003^b^

Abbreviations: ASA, American society of anesthesiologists; EBL, estimated blood loss; ICU, intensive care unit; IQR, inter quartile range; LOS, length of stay.

^a^ Wallis test.

^b^ chi‐square test.

### 3.1 Independent Predictors of Adverse Outcomes

To complement the team‐based analysis, we performed multivariate modeling to identify patient‐ and procedure‐level factors associated with adverse outcomes.

### 3.2 High Clavien–Dindo Classification (Grade > 2)

Patients who underwent upper gastrointestinal (GI) surgery had the highest risk of developing severe postoperative complications, with a hazard ratio (HR) of 8.31. Lower GI surgery was also associated with increased risk (HR 2.56), whereas breast surgery significantly reduced the likelihood of severe complications (HR 0.13). Additional factors associated with increased risk included an operative time exceeding 180 min (HR 2.97), estimated blood loss of ≥ 500 mL (HR 2.39), a preoperative ASA physical status classification of ≥ 3 (HR 3.69), and undergoing emergency surgery (HR 2.27). All of these associations were statistically significant (Table 2).

**TABLE 2:** Multivariate analysis between risk factors, high clavien dindo (grade > 2) classification, in hospital death and mortality (*n* = 1123).

Risk factors	High clavien dindo		In hospital death		Mortality	
	HR (95% CI)	*p* value	HR (95% CI)	*p* value	HR (95% CI)	*p* value
Female	—	—	0.24 (0.06, 0.99)	0.031	—	—
Type of surgery Upper GI Lower GI Breast Neck Soft tissue	8.31 (4.08, 16.93) 2.56 (1.26, 5.17) 0.13 (0.02, 1.03) — —	< 0.001 0.008 0.01 — —	6.24 (1.82, 21.43) — — — —	0.003 — — — —	2.51 (1.37, 4.57) — 0.09 (0.03, 0.25) 0.12 (0.03, 0.57) 0.21 (0.04, 1.01)	0.003 — < 0.001 < 0.001 0.023
Malignancy	—	—	—	—	9.78 (4.95, 19.3)	< 0.001
Laparoscopic surgery	—	—	—	—	0.44 (0.23, 0.85)	0.035
Operative time > 180 min	2.97 (1.64, 5.36)	< 0.001	—	—	0.5 (0.26, 0.94)	0.027
EBL ≥ 500 mL	2.39 (1.07, 5.32)	0.036	—	—	—	—
ASA ≥ 3	3.69 (2.15, 6.32)	< 0.001	20.67 (2.57, 166.19)	< 0.001	5.2 (2.97, 9.11)	< 0.001
Emergency surgery	2.27 (1.26, 4.08)	0.007	12.1 (2.88, 50.94)	< 0.001	2.58 (1.38.4.8)	0.003
Co‐surgeon	—	—	3.65 (1.06, 12.63)	0.041	—	—
Less than 3 years surgical practice	—	—	—	—	2.73 (1.59, 4.71)	< 0.001

*Note:* Surgical category definitions: Upper GI = esophagectomy, gastrectomy, EGJ resection, fundoplication; Lower GI = colectomy, low anterior resection, anorectal surgery; Soft tissue = sarcoma resection, large lipoma excision; Neck = thyroidectomy, neck node dissection, parathyroidectomy; Breast = mastectomy, breast conserving surgery, axillary surgery.

Abbreviations: ASA, American society of anesthesiology; CI, confidential interval; EBL, estimated blood loss; HR, hazard ratio.

### 3.3 In‐Hospital Mortality

In‐hospital death was significantly less likely in female patients (HR 0.24), suggesting a protective effect. Conversely, upper GI surgery was associated with a markedly higher risk of death during hospitalization (HR 6.24). Patients with an ASA score of ≥ 3 had a dramatically elevated risk (HR 20.67), as did those undergoing emergency surgery (HR 12.1). Interestingly, the presence of a co‐surgeon during the operation was also linked with higher in‐hospital mortality (HR 3.65), possibly reflecting case complexity. All findings were statistically significant (Table 2).

### 3.4 Overall Mortality

Several risk factors were identified for increased long‐term mortality. Upper GI surgery was associated with more than double the risk of death (HR 2.51), while lower GI (HR 0.09), breast (HR 0.12), and neck (HR 0.21) surgeries were associated with reduced risk. Malignancy emerged as a strong predictor of mortality (HR 9.78), as did a high ASA score (HR 5.2), emergency surgery (HR 2.58), and limited surgical experience (< 3 years; HR 2.73). On the other hand, laparoscopic surgery was associated with a protective effect (HR 0.44). Notably, prolonged operative time (> 180 min) was linked with lower overall mortality (HR 0.5) (Table 2).

## 4 Discussion

This study aimed to evaluate whether different surgical team configurations during a surgeon's early career impact perioperative outcomes. The S + S group, despite managing higher‐risk cases, allowed for mentorship in complex procedures. The S + J group demonstrated a balanced structure combining supervision and hands‐on experience. Surgeries performed by the S + S group were associated with longer operative times, greater blood loss, and higher complication rates likely reflecting the complexity of these high‐risk cases. Although such collaboration can enhance precision, it may also introduce inefficiencies due to shared decision‐making and procedural complexity. When compared with previous studies, our findings align with research indicating that high‐risk, complex surgeries typically require more extensive intervention and are associated with prolonged operative times and increased complication rates [1]. Our observation that solo surgeons managed less complex cases is consistent with prior studies suggesting that experienced surgeons optimize efficiency and minimize intraoperative risk when working independently [3]. Additional evidence supports that experienced surgeons tend to have shorter surgical durations and reduced blood loss, resulting in better clinical outcomes [14, 15].

The observed differences in outcomes, such as prolonged operative time and higher complication rates in the S+S group, likely reflect greater case complexity rather than the direct effect of senior assistance. Surgeries involving the S + J group demonstrated intermediate levels of operative time, blood loss, and complication rates, indicating a balance between training and patient safety. These findings emphasize the importance of structured mentorship, where junior surgeons gain hands‐on experience under guidance, fostering skill development without significantly increasing risk. Although procedures may be slightly prolonged, the presence of a more experienced surgeon helps maintain surgical quality and minimize complications, supporting both educational and clinical objectives [15]. In a systematic review, significant differences in operative time, blood loss, or complication rates were observed between tele‐mentored and directly mentored procedures [16]. Furthermore, performing mentorship can decrease the learning curve for complex procedures without compromising patient safety [17].

Differences in LOS across the groups likely reflect underlying case complexity. The S + S group had the longest LOS, likely due to managing more severe cases, whereas the S group demonstrated shorter stays, consistent with lower‐risk procedures. The S + J group showed intermediate LOS, highlighting the benefit of structured mentorship in balancing training and recovery outcomes. A systematic review also found that stable surgical teams were associated with reductions in operative time, complication rates, and LOS [18]. Additionally, it has been reported that both procedure complexity and team size significantly affect surgical duration. When procedure complexity and patient condition were held constant, the addition of one surgical team member increased procedure length by an average of 7 min [19].

Our findings showed that high‐grade Clavien–Dindo complications increased with surgical risk. Low‐risk surgeries had fewer complications, whereas high‐risk cases often involving complex procedures or medically fragile patients were associated with significantly higher complication rates [20, 21]. The first‐year surgeons exhibited the highest complication rates, suggesting that early‐career practitioners may face challenges with technical execution, intraoperative decision‐making, and postoperative care, thereby increasing the risk of severe complications [22]. These observations underscore the importance of robust early training and structured mentorship in reducing adverse outcomes. Complication rates declined after the first year, reaching their lowest point during the third year of independent practice. A slight increase in later years may reflect greater involvement in high‐risk or complex procedures, as surgeons assume more advanced roles including mentoring junior colleagues [23]. High‐risk surgeries inherently demand advanced skills, and the increased complication rates may reflect this dual responsibility. These findings support the need for continued mentorship and structured learning pathways to sustain skill development and optimize patient safety [20, 23].

Upper gastrointestinal (GI) and lower GI surgeries were associated with a significantly increased risk of severe postoperative complications, consistent with studies reporting higher morbidity for abdominal and oncologic procedures owing to anatomical complexity and physiological stress [21, 23]. By contrast, undergoing breast surgery was independently linked to a lower risk, mirroring the favorable profiles reported for minimally invasive and superficial procedures [1]. Intra‐operative factors (operative time > 180 min and estimated blood loss ≥ 500 mL) were also significant predictors of severe complications as Pasquer et al. who showed that prolonged operations and technical challenges directly amplify complication severity [16]. Likewise, ASA class ≥ 3 and emergency status remained strong predictors of high Clavien–Dindo grades, supporting earlier evidence that pre‐operative physiology and urgency drive surgical risk [2, 3]. Female patients experienced lower in‐hospital mortality as Oliver et al.’s findings that sex‐related physiologic and immunologic differences may aid recovery [15]. Upper GI surgery, malignancy, high ASA status and emergency surgery all increased overall mortality, in line with reports from oncologic and high‐risk cohorts [10, 21]. Malignancy showed the strongest association, underscoring disease biology over procedural factors. Limited surgical experience (< 3 years) also correlated with higher mortality, reinforcing the value of early mentorship and graduated autonomy [18, 22]. Laparoscopic surgery was independently associated with reduced mortality, aligning with global trends that favor minimally invasive approaches for selected surgical patients [17]. Interestingly, operative time > 180 min was associated with lower overall mortality; this inverse relationship likely reflects careful selection and optimization of longer elective procedures, where deliberate intra‐operative decisions can lead to better outcomes [5].

A key strength of this study is its large sample size, enabling robust comparisons across surgical teams and collaborative models. The study offers a comprehensive view of how experience influences outcomes. However, the retrospective design limits causal conclusions. We could not pair‐match individual procedures by complexity. Although adjusted analyses were performed, unmeasured differences may still influence outcomes. Selection bias and unmeasured variables may have affected results. Mentorship is vital but should be carefully managed in complex cases. These findings may be less generalizable to healthcare systems in which surgeons are subspecialized early in their careers, as mentorship structures and learning curves may differ significantly. Cross‐system comparisons may further clarify institutional impacts. Future prospective studies are needed.

## 5 Conclusion

The first 3 years of surgical practice represent a critical transition, with complication rates peaking in year one and improving thereafter. However, increasing case complexity in later years reintroduces risks, particularly in upper GI surgery, emergency cases, high ASA scores, malignancy, and limited experience (< 3 years). Junior‐assisted models showed balanced outcomes, supporting structured mentorship and stepwise case exposure as essential strategies for achieving safe surgical autonomy.

## Author Contributions

**Wongsakorn Chaochankit:** conceptualization, methodology, writing – original draft, writing – review and editing, investigation, data curation, validation, formal analysis. **Seechad Noonpradej:** funding acquisition, visualization, resources, project administration, writing – review and editing. **Srila Samphao:** supervision, resources, writing – review and editing, visualization, software. **Somrit Mahattanobon:** project administration, writing – review and editing, supervision, resources, visualization. **Chutida Sungworawongpana:** conceptualization, investigation, methodology, validation, supervision, resources, writing – review and editing, writing – original draft.

## Conflicts of Interest

The authors declare no conflicts of interest.

## Supporting information

Table S1

## Data Availability

See Table S1.
