# Supplementary material for: The Critical Transition in Surgical Experience: Impact of the Early Years of Surgical Practice on Perioperative Outcomes and Team Collaboration
Source: World J Surg. 2025 Aug 1;49(9):2436–42. doi: 10.1002/wjs.70030 (PMC12435602; doi:10.1002/wjs.70030)
Supplement: Supplementary file 1 — Table S1 [file WJS-49-2436-s001.docx]

**Supplement table 1 Relationship between high Clavein–Dindo classification (more than grade 2), surgical risk, surgeon collaboration, and early surgeon experience^#^**

| Year | Low risk | | | Intermediate risk | | | High risk | | | Total |
| --- | --- | --- | --- | --- | --- | --- | --- | --- | --- | --- |
|  | S | S+S | S+J | S | S+S | S+J | S | S+S | S+J |  |
| First |  |  |  | 6 | 2 |  | 1 | 4 |  | 13 (12.6%) |
| Second |  |  |  | 7 | 1 |  | 4 | 1 |  | 13 (7%) |
| Third | 3 |  |  | 8 | 2 | 2 | 1 | 4 | 1 | 21 (11.3%) |
| Fourth | 1 |  |  |  | 1 | 2 | 1 |  |  | 5 (2%) |
| Fifth | 5 |  | 2 | 9 | 1 | 2 | 2 | 3 | 3 | 27 (8%) |
| Sixth | 1 |  |  | 3 |  |  |  | 1 |  | 5 (6%) |
| Total | 10 | 0 | 2 | 33 | 7 | 6 | 9 | 13 | 4 | 84 |
|  | 12 | | | 46 | | | 26 | | |  |

= zero.

# p value = 0.001
